# Experiences and wellbeing of Samaritans crisis line volunteers in Ireland during the COVID-19 pandemic: A qualitative study

**DOI:** 10.1177/00207640221089538

**Published:** 2022-04-19

**Authors:** Aoife Cooney, Darragh McCashin

**Affiliations:** School of Psychology, Dublin City University, Ireland

**Keywords:** Crisis line volunteers, Samaritans, COVID-19, pandemic, wellbeing, thematic analysis

## Abstract

**Background:**

The literature demonstrates that working in a crisis line volunteer role, despite being rewarding, poses a risk of adverse effects on health and additional pressure exists with the COVID-19 pandemic. Despite this, there is limited research on the experiences of such volunteers.

**Aims:**

This study aimed to explore the experiences of Samaritans volunteers in Ireland during the COVID-19 pandemic, thereby contributing to the overall knowledge of crisis line volunteering, and gaining an understanding of how this role is impacted by the pandemic.

**Method:**

A qualitative approach was taken, and semi-structured interviews were conducted with volunteers from Samaritans branches in Ireland (n = 13). Data were then analysed using thematic analysis.

**Results:**

Four overarching themes were identified: (1) responding to calls in the context of COVID-19, with sub-themes being change in the nature of calls, reliance on existing approach in handling calls and varying emotional responses to calls, (2) sense of loss, with sub-themes being loss of older and vulnerable volunteers, reduced social connectedness and restricted aspects of the service, (3) positive experiences, with sub-themes being supportive culture, new personal skills and perspectives and volunteering as an escape from lockdown and (4) adaptation challenges, with sub-themes being logistical changes and concern for sustainability of the service.

**Conclusions:**

Findings highlight important insights into the experiences of Samaritans volunteers in Ireland, revealing that the pandemic brought about challenges, but also gave rise to some positive experiences. Implications of these findings are discussed in the context of the existing literature and recommendations made.

## Introduction

### Crisis line volunteering

Telephone crisis line services provide valuable support which is accessible and free to people experiencing emotional distress, relieving pressure on state-provided mental health systems (Hoffberg et al., 2020; Sundram et al., 2018). Such services are typically provided by volunteers in non-profit organisations who are generally not required to hold a professional qualification (e.g. Samaritans in Ireland and the UK, Lifeline in Australia and Suicide Écoute in France). The literature presents positive and negative aspects of the crisis line volunteer role. On one hand, findings echo those from research into volunteering in general, with volunteers experiencing improved wellbeing and life satisfaction (Jenkinson et al., 2013; Yeung et al., 2018). Benefits of crisis line volunteering specifically include satisfaction from helping others, social connectedness with fellow volunteers and personal development of perspectives and skills (Hector & Aguirre, 2009; Mishara & Giroux, 1993; Praetorius & Machtmes, 2005; Smith et al., 2018; Sundram et al., 2018).

However, mental health work can also have negative effects on the service provider’s own wellbeing (Arnold et al., 2005; Cummins et al., 2011). Crisis line volunteers encounter a wide range of intense, unpredictable topics and the anonymous nature of the service leads to a lack of control and uncertain outcomes (Lamb & Cogan, 2016; Vattøe et al., 2020; Willems et al., 2020). Additionally, abusive calls are a significant cause of upset contributing to dropout (Middleton et al., 2014; Pollock et al., 2010). Two recent systematic reviews on crisis line volunteer wellbeing (Kitchingman et al., 2017; Willems et al., 2020) conclude that this population is at risk of declined psychological health, experiencing symptoms of stress, burnout, compassion fatigue and vicarious traumatisation.

Implications pertain not only to volunteer health, but also to the quality of service provided (Johnson et al., 2018). Yet, the literature has tended to focus on the callers’ experience, rather than the volunteers’ (Hoffberg et al., 2020; Howlett & Collins, 2014) and both systematic reviews highlight the dearth of research examining the effects of the crisis line role on individual volunteer wellbeing (Kitchingman et al., 2017; Willems et al., 2020).

### The context of this study: Samaritans

A prominent crisis line organisation which has influenced the development of telephone helplines worldwide (Mishara et al., 2016), the Samaritans was established in 1953 and volunteers provide 24/7, free and confidential emotional support to callers from over 200 branches across the UK and Ireland (Samaritans, n.d.), mainly by telephone, as well as through email, face-to-face contact and by community outreach. Various studies have concentrated on the Samaritans organisation, however, a significantly smaller proportion of these have focused specifically on the volunteers themselves. In an independent evaluation of the organisation, volunteer experience was described as generally positive, suggesting it is a rewarding, fulfilling role associated with personal growth and camaraderie with other volunteers (Pollock et al., 2010). However, associated challenges were also highlighted, including abusive calls and uncertainty about the handling of calls. Subsequent research presents similar findings: lack of control and doubt exist as stressors, while the social connections and organisational structure and support are important to successful coping, with avoidant coping predictive of negative health outcomes (Lamb & Cogan, 2016; Roche & Ogden, 2017). Most recently, Smith et al. (2018) qualitative exploration of UK Samaritans’ experiences suggest that while taking on additional roles within the organisation contributes to a sense of belonging, multiple responsibilities can be a source of stress, with an obligation felt to take on more.

While these findings provide important insight into the Samaritans volunteer experience and demonstrate a similar pattern to that of findings on crisis line volunteering in general, this research is limited by its narrow focus on UK volunteers only and some are older findings (e.g. Lamb & Cogan, 2016; Pollock et al., 2010).

### The impact of COVID-19

Further, additional pressure has since been placed on crisis line volunteers with the ongoing coronavirus (COVID-19) pandemic, an unprecedented public health challenge. Emerging research has raised concerns of effects on population mental health, specifically the development of new mental health issues and an exacerbation of existing ones (Holmes et al., 2020; Ramiz et al., 2021; Van Bavel et al., 2020; Yao et al., 2020). In Ireland, anxiety and depression are common experiences during the pandemic (Hyland et al., 2020), with ‘a huge escalation of mental health need’ anticipated post-pandemic (O’Connor et al., 2021, p. 105). Disruptions to mental health services means an increased demand on crisis line services (Wind et al., 2020), with the Health Service Executive (HSE) in Ireland providing contacts for remote support services, including the Samaritans, in lieu of face-to-face resources (Health Service Executive [HSE], 2020). Turkington et al. (2020) analysis of calls to Samaritans in Ireland during the pandemic observed an increase in the number of calls and their duration, attributing this to loneliness, exacerbation of existing mental health issues and limited access to other support. The authors also specify the need to re-evaluate services and provide appropriate training and support to volunteers.

Significant research has been conducted with healthcare and mental health professionals, exploring the effects of the pandemic on their wellbeing and finding notably high levels of distress, depression, anxiety and burnout in comparison to the general population’s (College of Psychiatrists of Ireland, 2020; Joshi & Sharma, 2020; Kelly, 2020; Vizheh et al., 2020). Less is known about the strain felt by crisis line volunteers. Joshi et al. (2020) found feelings of helplessness and powerlessness among helpline counsellors in India, as well as aspects of calls paralleling their own experiences during the pandemic, such as uncertainty, fear of the virus and separation from loved ones. However this was a sample of professionals rather than volunteers. Bearing in mind that crisis line volunteers are also experiencing the general effects of the pandemic, along with increased pressure in an already demanding role, research on the wellbeing of this population is clearly warranted.

### Rationale for present study

A gap exists in the research, with a paucity of studies examining crisis line volunteers (Kitchingman et al.,2017; Sundram et al., 2018; Willems et al., 2020). Studies which have sampled Samaritans volunteers were restricted to UK volunteers only (e.g. Roche & Ogden, 2017; Smith et al., 2018); to our knowledge, no qualitative study has been carried out previously with Samaritans volunteers in Ireland. Moreover, there is an exigency to examine the experiences and health of those working to provide emotional support throughout the pandemic, given the significant challenge it poses. This present study therefore aims to address this need by exploring the experiences of Samaritans volunteers in Ireland during the COVID-19 pandemic. Insight into this can provide valuable knowledge pertaining to supporting the volunteers in their role and with their own wellbeing, which in turn translates into better service provision.

## Methods

### Design

A qualitative, exploratory research design using semi-structured interviews was employed; the aim was to explore the experiences of the volunteers and, as Braun and Clarke (2014) specify, qualitative research enables a richer description of participants’ own experiences and perspectives.

### Context

Established in Ireland in 1961, Samaritans currently has over 2,000 active volunteers operating 21 branches across the country. Volunteers are selected through application and interview and undergo comprehensive training for the role and supervision from a ‘mentor’ (an experienced volunteer) before taking calls on their own. On average, volunteers engage in listening duties in the branch for approximately 3 hours every week and volunteers can also hold other positions within the organisation.

At the time of interviews, March 2021, Ireland was under strict government restrictions in order to reduce virus transmission (see Department of the Taoiseach, 2020).

### Participants

A total of 13 participants completed interviews for this study (*n* = 13). This sample size was deemed appropriate for this study; Braun and Clarke (2013) recommend a sample size of 6 to 15 participants for a medium-sized study (e.g. a Master’s project) with an interview design employing thematic analysis (Terry et al., 2017). Participants ranged in age from 28 to 74 years (*M* = 56.92, *SD* = 14.38). Of these, 10 identified as female and 3 as male. Varying lengths of time volunteering with Samaritans were reported, from less than 3 to over 20 years. Of the 21 Samaritans branches in Ireland, volunteers from 10 participated in this study.

### Procedure

A letter outlining the study was emailed to the Regional Director of Samaritans Ireland, who agreed to circulate the recruitment advertisement among the Irish branches of the organisation. Volunteers were eligible if they had completed training and were actively volunteering with an Irish branch of the Samaritans. A total of 14 volunteers (self-identified as 11 females and 3 males) responded to the recruitment advertisement, and a detailed plain language statement and consent form were sent to these volunteers via email addresses they provided. One female volunteer then declined to take part, citing the interview design of the study as the reason. Individual semi-structured interviews with open-ended questions were conducted with participants via Zoom (Archibald, 2019) in March 2021, a year into pandemic restrictions in Ireland. Table 1 provides the fully detailed interview schedule, developed based on a literature review and pilot interviews conducted with two Samaritans volunteers, in line with recommendations (Kallio et al., 2016). Interviews ranged in length from 13 to 50 minutes, with the average duration being 27 minutes and were led by the first author. Interviews were recorded using the Zoom recording device and audio recordings were held on password-protected encrypted files.

**Table 1.** Semi-structured interview schedule.

Opening
1. Can you describe your role as a Samaritans volunteer for me please?
2. Have you any other roles within the Samaritans organisation?
Motivations
1. Can you tell me what motivated you to become a Samaritans volunteer?
2. Since becoming a volunteer has there been any change in your motivation, as in what motivates you to stay volunteering?
Benefits
1. What are the benefits that you find from volunteering?
2. What would you say has been the personal impact of being a volunteer on you, if any?
3. What does the Samaritans volunteer community mean to you?
Challenges
1. In general, what are the challenges you face being a volunteer, if any?
2. Have you ever felt inclined to take a step back, or a break from the role?
3. How do you deal with the challenges of the role?
Impact of COVID-19
1. What would you say has been the impact, if any, of the COVID-19 pandemic on your role as a volunteer?
2. Would you say that you have noticed a difference in the calls that you have received?
3. Do you find that you operate differently as a result of the pandemic?
4. Going forward, what do you think is the biggest challenge facing the Samaritans as a result of the ongoing pandemic?
5. If the Samaritans had unlimited resources, what do you think would be the ideal way to support the volunteers during the pandemic?
Closing
1. Is there anything that I haven’t asked you that you would like to discuss about your experiences?

Interviews were transcribed verbatim by the first author. To ensure anonymity, participant names were replaced with pseudonyms and any other identifying information, for example Samaritans branch location, were omitted.

### Data analysis

Reflexive thematic analysis was conducted on the data, in line with Braun and Clarke’s (2006, 2019) guidelines. With the focus of the study being on the perspectives and experiences of the volunteers, this approach was deemed fitting as it is in line with recommendations of its use in research (Braun & Clarke, 2013, 2014). Braun and Clarke’s (2006) recommended six-phase procedure was carried out, as presented in Table 2 (see Supplemental Material 1 for further details).

**Table 2.** Steps in data analysis.

Step 1: Familiarisation with data	Transcripts were read and re-read
Step 2: Initial coding	Dataset was coded to identify relevant keywords
Step 3: Identify themes	Codes were collated into potential themes and sub-themes
Step 4: Review of themes	Themes were reviewed and revised
Step 5: Define and name themes	Themes were appropriately defined
Step 6: Analysis of themes	Final analysis was compiled

An initial hand-coding took place, before the qualitative software package NVivo (release 1.5.1 (940) for Windows, QSR International, 2021) was used to manage the data. Data analysis was conducted by the first author. Code identification was informed by the literature review, but an overall inductive approach was taken to analysis.

### Ethics

Ethical approval was obtained on 20th January 2021 from the Dublin City University School of Psychology Ethics Committee.

### Reflexivity

In line with the standard for enhancing rigour and credibility in qualitative research, the practice of reflexivity was considered (Berger, 2015; Dodgson, 2019). The first author is a crisis line volunteer with a different organisation. While this allowed some prior knowledge of the subject and facilitated empathy during interviews, it was important to maintain an awareness of the researcher’s position to reduce the risk of bias. Participants were not known to the researchers, thus avoiding any risk of a researcher-participant relationship. To embed transparency, the COnsolidated criteria for REporting Qualitative research (COREQ; Tong et al., 2007) was utilised (see Supplemental Material 2).

## Results

Four overarching themes were identified: (1) responding to calls in the context of COVID-19, (2) sense of loss, (3) positive experiences and (4) adaptation challenges, with 11 sub-themes; Table 3 presents a summary of these.

**Table 3.** Summary of themes and sub-themes.

Theme	Sub-themes
1. Responding to calls in the context of COVID-19	(a) Change in the nature of calls
	(b) Reliance on existing approach in handling calls
	(c) Varying emotional responses to calls
2. Sense of loss	(a) Loss of volunteers
	(b) Reduced social connectedness
	(c) Restricted aspects of the service
3. Positive experiences	(a) Supportive culture
	(b) New personal skills and perspectives
	(c) Volunteering as an escape from lockdown
4. Adaptation challenges	(a) Logistical changes
	(b) Concern for sustainability of the service

**Figure 1.**
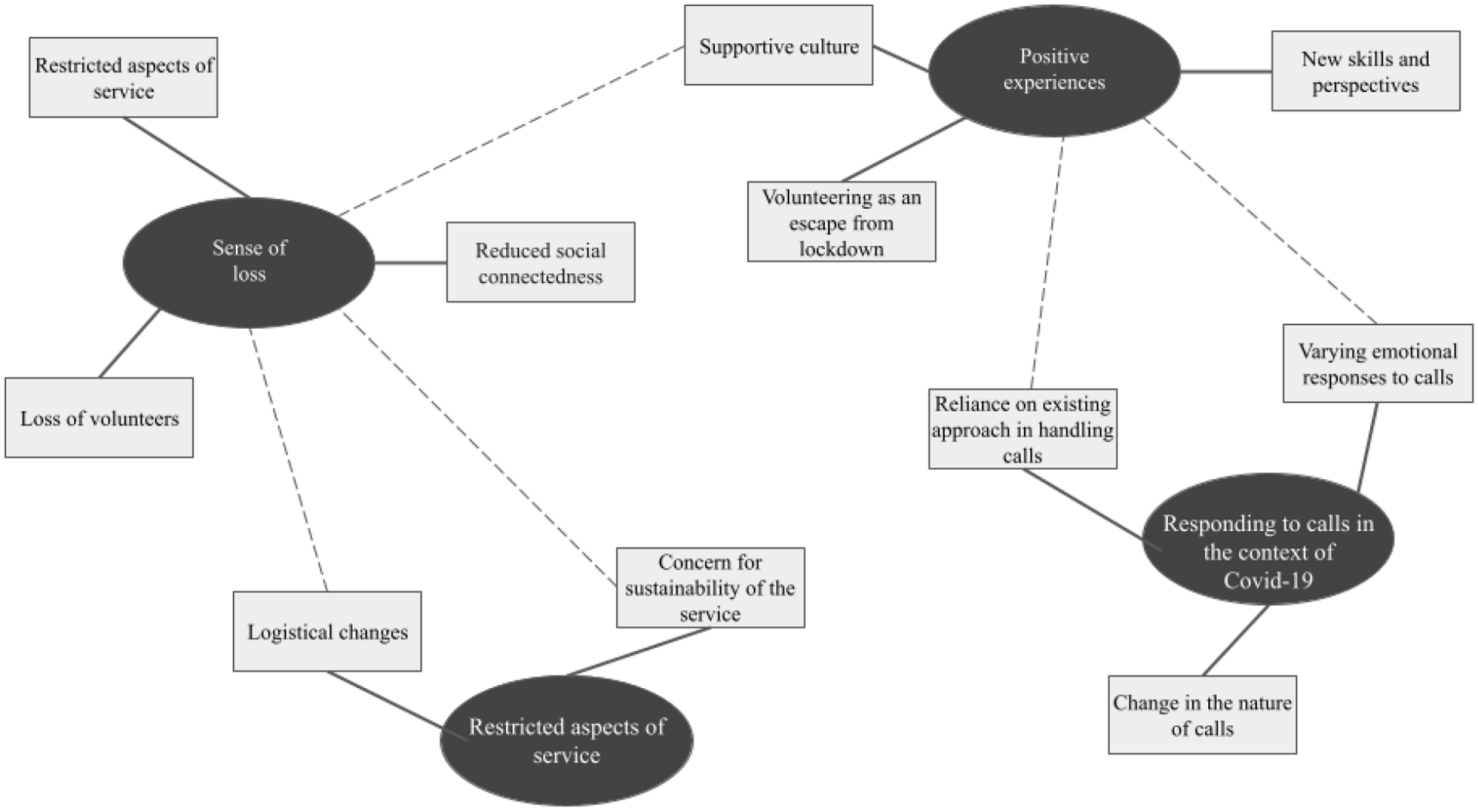
Final thematic map, with interconnectedness illustrated.

While these main themes are salient and distinct, it is important to note that some of the sub-themes are interwoven, as demonstrated in the final thematic map presented in Figure 1.

The following subsections expand on each theme and include illustrative quotations from participants. Some quotes have been edited to omit unnecessary repetition (e.g. ‘it’s-it’s’) or redundant words (e.g. ‘um’; Thorne, 2020). Omissions are denoted by an ellipsis in curved brackets (i.e. (. . .)).

### Responding to calls in the context of COVID-19

The first theme refers to the volunteers’ experiences of receiving and handling calls throughout the COVID-19 pandemic, with respect to the nature of the calls, how the volunteers approached the calls and the volunteers’ emotional responses.

#### Change in the nature of calls

Each volunteer spoke to some extent about the content of calls: change in the nature of the calls during the pandemic was identified, with COVID-19 described as being present, in some form, in almost every conversation: ‘COVID-19 (. . .) umbrellas kind of every conversation that we have now. (. . .) it mightn’t be a direct worry with the caller, but it certainly lurks in the background if it’s not in the foreground (John)’. Volunteers also noticed an exacerbation in existing issues for callers, attributing this to safety restrictions and worry. In particular, there was a perception that callers’ feelings of loneliness and isolation had increased:

Things are more magnified I suppose, issues are the same, you know, all life issues. From, isolation, loneliness, anxiety, depression, relationship problems, family problems, financial, health issues. Ah, they haven’t changed. They’re more magnified- isolation is probably stronger, I suppose, in terms of people who can’t get out and they can’t do activities. (Seán)

#### Reliance on existing approach in handling calls

Many participants believed that their approach to engaging with callers remained the same, feeling that initial Samaritans training had prepared them to support people in distress and that the topic of COVID-19 was seen fundamentally as another distress for the caller:

As a volunteer, the guidelines are still the same, that we listen to whatever the caller wants to tell us and we will listen and help them and encourage them to talk for as long as they want. So that in itself hasn’t changed. (Lily)

However, some volunteers also felt that the pandemic added a layer of difficulty when using pre-pandemic approaches: ‘There’s definitely more uncertainty so you kinda have to manage-manage that and kind of explore that with people, about the future and what they see it being but I wouldn’t say my approach is overly different’ (Cian).

Normally you would say to them, you know, ‘Have you any plans for later on today, you know, would you go for a walk, you know, you mentioned such a person before, would you meet them for coffee or something?’ (. . .) but all those things aren’t there at the moment. (. . .) It’s only made it- you just have to think a bit more. (Niamh)

#### Varying emotional responses to calls

Relating to their own response to calls, volunteers remarked on the different emotions which arose and the variance in these. It was clear that providing support for the callers brought about a positive emotional reaction in the volunteer when they felt that they had ‘been there’ for that caller: ‘Regardless of what type of call you get, because you’re at the other end of the phone, it’s very rewarding, full stop, regardless of the type of qualities. That you can be there for somebody’ (Chloe). However, certain calls were pinpointed as a challenge - namely distressing calls and abusive calls. Listening to others in distress can unsettle volunteers, with Alice stating ‘The calls you take and the things that you hear, they can be quite upsetting at times’. Seán expressed how disheartening it can be for volunteers to deal with people misusing the service with abusive calls:

We get challenging kinds of calls, our female volunteers get a lot of sex calls (. . .) people are coming in to do something and they’re getting abused, effectively. (. . .) at times you might come in and have a duty or two and it’s really, really, you know, crap, that the calls are not of any quality.

Hazel further outlined how such callers can be ‘very aggressive, very unpleasant, manipulative in a different way who appear to be trying to completely undermine your self esteem’, exemplifying the extent of this issue. Additionally, many volunteers described a change in how calls affected them emotionally during the pandemic. John noted ‘you are on a level playing field with callers when it comes to COVID-19’. This aspect served as both a positive and negative for volunteers. Experiencing the pandemic themselves enabled volunteers to better empathise with the callers:

There’s a lot of things we haven’t gone through in life and we don’t have that understanding and we try to get that from the person we’re talking to, to get a better understanding of how they’re feeling about something like that and how it has impacted on their lives, but having gone through the thing of COVID and seeing the isolation and loneliness, yeah without a doubt, it gives you a better understanding of what’s happening. (Megan)

However, it was clear that COVID-19-related calls resonated with volunteers and brought about difficult emotions, with conversations hitting closer to home for some:

COVID-19 is affecting everybody. Of course, it is, in-in big and small ways. And, you know, it does make it that little extra tough to be really positive and rock solid going into a duty. (. . .) if a caller wants to give out yards about COVID-19 and how this and that and their lives are now gone. You know, you can’t join them in that. Although you-you might want to (laughs) (John)

One participant described how listening to older callers ring in with isolation or loneliness worries made them think of their own situation:

When it’s very close to home, especially during COVID, an awful lot of the older people ringing that are isolated, you know, they’re not obviously seeing anyone, haven’t seen family for, you know, for a year or so now. Ahmm, and then it’s sort of, you know, that’s my situation (. . .) that’s the challenge of it, it sort of makes you-, you know, things that are going on in your life as well. (Jodie)

### Sense of loss

The majority of volunteers spoke about the sense of loss felt in their role during the pandemic. Three sub-themes represent this finding: loss of volunteers, reduced social connectedness and restricted aspects of the service.

#### Loss of volunteers

The decline in volunteer numbers due to restrictions, resulting in gaps in shifts, was perceived to have a major impact on all volunteers. Often extra shifts were taken to ensure calls were answered and there were mixed experiences regarding this, with some finding it challenging:

Because of the over-seventies cocooning it’s harder to fill the slots. So I ended up working at more than one shift a week for a lot of weeks. That can be tiring and draining (. . .) and there’s no magic wand, the centre still has to be filled. So a lot of people would end up doing extra shifts and that can be tiring. (Chloe)

On the other hand, many saw it as their duty to step up and take on extra shifts to ensure that the service was maintained, with Julia stating: ‘Those of us who are able to do duty feel that’s a privilege, you know, it- we can do it, so we do it’. Cian felt that the pandemic ‘pushed (them) a little bit more to my duties’. Indeed Jodie had been on a break from volunteering, but was motivated to return to the role: ‘When people had to cocoon at the beginning of COVID, I stepped up back then (. . .) during COVID it’s definitely put things in perspective and there is a real need for doing, you know, what we do’. The loss of volunteers was also a significant challenge for those in director roles: ‘In my role as (. . .) director, it’s been hugely challenging because we lost nearly fifty percent of our volunteers in the first part of COVID, mainly because we have quite an older population’ (Seán).

#### Reduced social connectedness

It was evident that restrictions had given rise to a loss of social interaction amongst the volunteers. The Samaritans community was described as important, with a sense of camaraderie: ‘You’re part of a team, so I get something out of it too like I- you know, you meet people, (. . .) I kind of enjoy that part’, (Alice). However effects of restrictions on connecting with fellow volunteers was heavily emphasised by all, with a sense of separation and distance prevalent which affected both the social aspect of the role and the support between volunteers:

We are a little fractured at the moment, as a community, because there’s-there are only ever two people allowed in the building at once. And the way it’s time slotted, volunteers have to leave and fifteen minutes later, another two volunteers will commence, so we never cross paths (John)

If you know your fellow volunteer has a difficult call or, say if I had a difficult call. Usually when they- when the other person would be finished the call they’d leave the phone off so that they’d be there to give you support immediately, or sometimes you may need them to listen in to the call, ehm, because of the content. And, so that is one impact, really, you know, although the support is there, it’s not- it’s not physically beside you, the way it normally would be. (Jacqueline)

This loss was also present in the running of the branches, with those volunteers holding director and deputy director positions mentioning the impact felt with respect to working with others:

We as a team have never met face to face (. . .) So you lose- there’s a lot lost in that sense. So yeah, that’s what we’ve lost, big time you know and it’s- yes, we’re very supportive of one another, but you’ve lost that initial contact of, ahm, of being there for one another. (Megan)

#### Restricted aspects of the service

This sub-theme captures the losses felt as a result of restrictions on service provision. The loss of fundraising was a cause for concern for directors, who translated it into a loss of income: ‘That obviously is an issue and for some branches that would be far more serious than for others, just depending on how they fund themselves’ (Julia). However, one branch director described this loss as both a positive and negative experience:

If anything (laughs) there’s less pressure (. . .) and less emails and there’s less things, because there’s not as much happening. But (. . .) there’s lots of things we can’t do, which we would have normally done because our fundraising would have been publicity at the same time, so, d’ya know, but we can’t do anything like that but in-in the same time we’re not worried about getting permits to do the fundraising. So, you know, kind of a two-edged sword. (Lily)

### Positive experiences

Despite the prevailing sense of change and loss, it was evident that volunteers also found positive experiences through their role during the pandemic, as exemplified by the three sub-themes of a supportive culture, new skills and perspectives and volunteering as an escape from lockdown.

#### Supportive culture

All of the volunteers related how supportive and positive the Samaritans organisation is, from both an organisational perspective and an interpersonal volunteer perspective. As noted, the connections between volunteers is a salient part of the role and this contributed to the sense of support, both as a Samaritan and as an individual outside of the role, with the Samaritans community referred to as ‘a little family’ (Rebecca):

You’d argue it’s a family anyway, but I mean you-you know that they’re there supporting you and you know they have your back(. . .) if you go into anything, highs, lows, or anything they will be there to cheer you on. (Lily)

With respect to the organisational culture, volunteers felt looked after, with the Samaritans support system identified by most as their main way of coping with distressing calls:

I would deal with them within-within the system, I talk about them with my- whoever was on duty with me, or the leader. Ahm, yeah- no, no the whole idea is to kind of be supported within Samaritans, because everybody understands what the calls are like, that’s-that’s the way the structure works and I think it works very well. (Julia)

This sense of a supportive organisational culture continued with the pandemic and in spite of the challenges faced, volunteers described feeling supported in the branches. This was in relation to physical safety, with Jacqueline noting ‘All the safety measures are in place, it’s probably one of the most sanitised buildings I’d say in (. . .) at the moment, really (laughs)’, as well as emotional support:

The support that I feel going into the place and how safe I feel and, eh, how considered I feel as well. You know, it was very clearly drilled into us that if we ever did feel uncomfortable going into the branch or anything like that, for whatever reason, you know, it’d be completely understood. (John)

#### New personal skills and perspectives

Positive experiences were reported in relation to personal growth through the volunteer role. The skills learned through the Samaritans training and the experience with callers were considered by many to be transferable and advantageous in their own lives: ‘My listening skills improved a thousand percent, um, I find that I use the Samaritans training in everyday events, to be non judgemental, it really helps me in my personal capacity’ (Chloe). Additionally, volunteers noticed how their role facilitated a change in their perspective, led them to be ‘more aware’, ‘more open-minded’ and have ‘a broader outlook on life’. Alice outlines the substance of this experience:

It just changes you intrinsically, it just changes how you look at things, it changes how you think, it makes you aware of how different things could be, how easy things can change for you in your life (. . .) personally it changed how I see things.

Volunteers also reflected on the benefit to their perspective during the pandemic:

Particularly through lockdown I found myself that one of the massive advantages is kind of recentering your focus, when you think you’ve had a pretty poor day and life is kind of bringing you down a bit, you go and do a duty, you hear some of the stuff that people are going through and some of the difficulties that people have and it really makes you appreciate what you have. (Cian)

#### Volunteering as an escape from lockdown

Despite the aforementioned reduced social interaction, the opportunity to engage with others, albeit in a limited capacity, was recognised as important:

Especially during COVID, from a selfish point of view, it’s been great because (. . .) I can go and do my duty and so I’m sort of getting out and I’m seeing people. (Jodie)

Indeed, providing the support to those callers feeling affected by the pandemic, often with respect to loneliness and isolation as mentioned, also provided the volunteers themselves with a means of preventing potentially similar effects:

It’s this isolation and loneliness. But from the point of view of even going in to see it and be there, it actually took that away from you, because you were actually doing something, the isolation and loneliness. So whereas you’d have possibly been impacted by it, it actually took it away, because you were actually going in there and being there. (Megan)

Hazel described their experience ‘cocooning’ where they haven’t been able to carry out their duties, or indeed be otherwise out and about. Their additional roles as a duty leader and in caller care, which could be carried out remotely from home, provided an opportunity for connectedness and engagement:

I think it’s done a lot for my mental health, it’s kept it, very much kept it- particularly during COVID, if I wasn’t doing this, I would definitely- I mean I do have some other interests as well, but it was such a good structure, it gives- and communicating with other people and feeling like doing something useful. (Hazel)

### Adaptation challenges

The final theme encapsulates the challenges faced by volunteers in the adaptation of the organisation and elaborates on their experiences of practical and logistical adjustments in branches, as well as their perspectives on the future impacts of the ongoing pandemic.

#### Logistical changes

Many volunteers mentioned the adjustments with restrictions in place, primarily the move from direct personal contact to online. This affected aspects such as recruitment interviews and training and was highlighted as a challenge, in particular by participants who hold recruitment and director roles:

That was actually quite difficult, that was maybe a challenge, eh, that I could say as well, was interviewing over the phone because usually face-to-face, it’s much easier interview to gauge people, you feel more comfortable as well, it’s quite hard over the phone. (Cian)

I noticed in my role of volunteer care that people often got stressed about the fact of having to do online training rather than face to face training. It didn’t bother me at all, but I know some people, it did. (Chloe)

One participant expressed concern for the older demographic of volunteers and the challenge the increased use of technology poses to them:

I think they’re not always taking sufficient account of the demographics of the-of the volunteers they have. People over- people who haven’t grown up with, um, IT, or haven’t used it in their working lives (. . .) not everybody in Samaritans is as keen on or as comfortable with the language and IT as they’d like to think. (Hazel)

#### Concern for sustainability of the service

The final sub-theme outlines how, based on their experiences, volunteers are worried about the future. Many expressed their fear of the ‘fallout’ of the pandemic with respect to mental health and the subsequent impact that would have on the Samaritans. Additionally, volunteers expressed a worry as to whether the organisation would be able to maintain the service, with an expected increase in demand meaning a need for more volunteers:

The fact that, you know, mental health has been pressurised by what’s going on is going to have an impact we’re not even aware of and I think our service, we’re-we’re needed, whether we have the capacity to manage that. (Cian)

More volunteers, a lot more are needed. And it’s to retain volunteers, retention. It’s-there’s no magic wand, because, especially nowadays with movement of people, you know, people move for different jobs or their lives change, especially now with the pandemic, so that’s a problem, too, for retention. (Chloe)

## Discussion

This study aimed to gain an understanding of Samaritans volunteers’ experiences in Ireland during the COVID-19 pandemic, using a qualitative approach. Key findings suggest that the pandemic has had a notable impact on the volunteer experience in a number of ways, from both a negative and positive perspective. Four overarching themes were identified from thematic analysis: (1) responding to calls in the context of COVID-19, (2) sense of loss, (3) positive experiences and (4) adaptation challenges. The following section will discuss these themes, which often intertwine, in the context of the literature.

### Responding to calls in the context of COVID-19

In keeping with prior research, calls were observed to have the potential to elicit a positive or negative emotional response in volunteers, highlighting the spectrum of emotions associated with the role (Kitchingman et al., 2017; Lamb & Cogan, 2016; Middleton et al., 2014; Pollock et al., 2010; Smith et al., 2018; Willems et al., 2020). The volunteers spoke about the changing nature of calls during the pandemic, mirroring trends in other crisis line organisations (Arendt et al., 2020; Batchelor et al., 2021; Joshi et al., 2020). Perceptions of an exacerbation of callers’ issues reflect Turkington et al. (2020) quantitative analysis of Samaritans calls and support emerging research surrounding the adverse effects of COVID-19 on population mental health (Holmes et al., 2020; Hyland et al., 2020; Yao et al., 2020).

This theme also highlights the duality of an increased sense of empathy in crisis line work, arising from first-hand experience (Gerace et al., 2015; Hodges et al., 2010). While volunteers better understand what callers are feeling, some felt that conversations arose which strongly resonated with them and, while the strength of the Samaritans training model was highlighted, this perceived parallel between volunteers’ own experiences and conversations presented a challenge. Similarly, Joshi et al. (2020) reported distress among professional crisis line counsellors from hearing stories similar to their own experiences during COVID-19. Previously, differences have been noted between professional counsellors and crisis line volunteers with regards to dealing with difficult topics (e.g. Lamb & Cogan, 2016). This alignment between present findings and recent work (Joshi et al., 2020) would indicate however that there is a similarity, at the very least in the context of crisis line work during the pandemic.

### Sense of loss and positive experiences

The themes and sub-themes identified are often interwoven and, when taken together, create an overall understanding of the volunteer experience. Discussing the themes ‘sense of loss’ and ‘positive experiences’ together reflects the sense of the pandemic being a double-edged sword for volunteers. With the loss of volunteer numbers came a prevalent sense of duty among the other volunteers to work extra shifts. Interestingly, this finding contrasts to previous assertions of feelings of obligation and stress in taking on more responsibility within the Samaritans (Lamb & Cogan, 2016; Smith et al., 2018). This discrepancy can potentially be understood in the context of the pandemic and the aforementioned increased empathy with experience, with this postulation further strengthened by the finding that a sense of relief was present where demands in directing roles were alleviated. This suggests that the sense of obligation observed in previous studies is associated with non-COVID-19 extra pressures.

In line with previous findings, volunteers outlined aspects of the role beneficial to their wellbeing, with respect to personal development and social connectedness (Hector & Aguirre, 2009; Lamb & Cogan, 2016; Mishara & Giroux, 1993; Praetorius & Machtmes, 2005; Smith et al., 2018; Willems et al., 2020). Indeed, the Samaritans community was described as ‘like a family’ and imperative to a supportive environment and successful coping. While this community was negatively affected by the decrease in social interaction during the pandemic, a silver lining was present: engagement in the volunteer role served as a preventative measure against the sense of loneliness that was prevalent amongst callers, benefitting volunteer wellbeing.

### Adaptation challenges

The fourth theme, adaptation challenges, underlines the difficulties faced in the adaptation of the organisation to operate during the pandemic. Primarily noted was the transition from face-to-face to online contact, particularly for those in additional roles and older volunteers. Research highlights similar transitional challenges during the pandemic in Irish mental healthcare services (Usman & Fahy, 2020). Moreover, volunteers expressed concerns of the long-term impacts of the pandemic with respect to adverse effects on population mental health and, subsequently, increased pressure on the service, volunteer numbers and volunteer wellbeing. Retrospectively, research shows serious effects on mental health and support services after a pandemic (Tzeng et al., 2020). Both sub-themes provide useful direction for the Samaritans going forward.

### Study strengths and limitations

To our knowledge, this is the first qualitative study focusing on the experiences of Samaritans volunteers in Ireland. As such, this research provides an insight into volunteers in an Irish context which is comparable to the existing knowledge of UK Samaritans volunteers. Additionally, this study builds upon the recommendations of previous work (Smith et al., 2018) in that participants were recruited from multiple branches rather than a single location, serving to capture a range of experiences across different branches and present a more comprehensive understanding.

Regarding limitations, female volunteers outnumbered male volunteers in this study (10:3). While, in general, more females volunteer than males in Ireland (Volunteer Ireland, 2016), Samaritans demographic data could not be identified. Given that the first author conducted all of the interviews and was the sole coder, there is also the risk of researcher bias. This was attempted to be mitigated by taking a reflexive approach, however future research would benefit from enhanced inter-rater reliability.

### Implications

Findings have a number of useful implications, both theoretically, with respect to understanding crisis line volunteering during the COVID-19 pandemic and practically, in terms of the Samaritans organisation in Ireland. A notable finding is the uncertainty experienced by volunteers when answering COVID-19-related calls. While volunteers fell back on their training and the usual guidelines, challenges arose in two aspects: doubt in exploring with a caller and distress where calls resonated with the volunteer’s experience. The Samaritans organisation should therefore be mindful of this and would perhaps benefit from providing supplementary training to counteract these challenges. Relatedly, such training could also be an opportunity for additional social interaction among volunteers (in keeping with guidelines). Taken together, these may help support a positive and healthy volunteer experience. An emphasis may also be necessary on training for older Samaritan volunteers who do not feel comfortable with technology use. The experience of these volunteers can be improved with continued support to reduce the apprehension of unfamiliar technology, as outlined by Lee (2014).

Nonetheless, the Samaritans organisational structure appeared to serve as a buffer for the volunteers throughout the pandemic in terms of coping and support and this finding has implications for other crisis line organisations, who can perhaps be guided by the Samaritans’ model. Turning to long-term implications, it is important from the perspective of both the volunteers and the callers that the Samaritans organisation is as prepared as possible to meet the anticipated demand. Future research may wish to further explore how crisis line organisations can effectively adapt to this novel context.

## Conclusion

The COVID-19 pandemic presented as a double-edged sword for Samaritans volunteers in Ireland, with positive and negative aspects. Findings add to the limited knowledge of the wellbeing of crisis line volunteers and provide insight into how this population has been affected by the pandemic. Given the unpredictable nature of the ongoing public health crisis and the considerable effects it has on population mental health and, subsequently, crisis line organisations, it is imperative that volunteers are sufficiently supported in meeting the anticipated increased demand. Future research should therefore aim to build upon these exploratory findings to expand the knowledge base and support the volunteers.

## Supplemental Material

sj-docx-1-isp-10.1177_00207640221089538 – Supplemental material for Experiences and wellbeing of Samaritans crisis line volunteers in Ireland during the COVID-19 pandemic: A qualitative study Click here for additional data file. Supplemental material, sj-docx-1-isp-10.1177_00207640221089538 for Experiences and wellbeing of Samaritans crisis line volunteers in Ireland during the COVID-19 pandemic: A qualitative study by Aoife Cooney and Darragh McCashin in International Journal of Social Psychiatry
