# Supplementary material for: Experiences and wellbeing of Samaritans crisis line volunteers in Ireland during the COVID-19 pandemic: A qualitative study
Source: Int J Soc Psychiatry. 2022 Apr 19;69(2):322–33. doi: 10.1177/00207640221089538 (PMC9983053; doi:10.1177/00207640221089538)
Supplement: sj-docx-1-isp-10.1177_00207640221089538 – Supplemental material for Experiences and wellbeing of Samaritans crisis line volunteers in Ireland during the COVID-19 pandemic: A qualitative study [file sj-docx-1-isp-10.1177_00207640221089538.docx]

**Supplementary Material 1:**

*List of codes grouped by theme and sub-theme*

| **Themes** | **Sub-themes** | **Codes** |
| --- | --- | --- |
| Responding to calls in the context of COVID-19 | Change in the nature of calls | Exacerbation of issues for  callers |
|  |  | COVID-19 mentioned |
|  |  | Unknown nature of calls |
|  | Reliance on existing approach in handling calls | Same approach to calls |
|  |  | Good training |
|  |  | Uncertainty in conversations |
|  | Varying emotional responses to calls | Rewarding |
|  |  | Sense of giving back |
|  |  | Continued motivation |
|  |  | Distressing calls |
|  |  | Better understanding |
|  |  | Empathising |
|  |  | COVID-19  calls resonating |
|  |  | Feeling affected by pandemic |
|  |  | Need to maintain boundaries |
|  |  | Emotional challenge to separate from calls |
|  |  | Need to be in good state of mind |
| Sense of loss | Loss of volunteers | Loss of older/vulnerable volunteers |
|  |  | Gaps in shifts |
|  |  | Taking on extra shifts |
|  |  | Sense of duty |
|  | Reduced social connectedness | Loss of social interaction |
|  |  | Social aspect to role |

**Supplementary Material 1:**

*List of codes grouped by theme and sub-theme (continued)*

| **Themes** | **Sub-themes** | **Codes** |
| --- | --- | --- |
|  | Restricted aspects of service | Loss of income |
|  |  | Less pressure |
| Positive experiences | Supportive culture | Support among volunteers |
|  |  | Shared vision |
|  |  | Variety of people |
|  |  | Organisational support structure |
|  |  | Never alone |
|  |  | Like a family |
|  |  | Feeling physically safe in branches during pandemic |
|  | New personal skills and perspectives | Change in perspective |
|  |  | Feel lucky in own life |
|  |  | Use of Samaritans training outside of organisation |
|  |  | Privilege to be able to provide support |
|  |  | Grow as a person |
|  |  | More aware as a parent |
|  |  | Improved listening skills |
|  |  | Skills complement career |
|  | Volunteering as an escape from lockdown | Opportunity to engage and interact during pandemic |
|  |  | Preventing loneliness during pandemic |
|  |  | Need to feel useful |

**Supplementary Material 1:**

*List of codes grouped by theme and sub-theme (continued)*

| **Themes** | **Sub-themes** | **Codes** |
| --- | --- | --- |
| Adaptation challenges | Logistical changes | Pressure felt by directors and deputy directors |
|  |  | Practical organisational adjustments |
|  |  | Challenge of increased technology use for older volunteers |
|  |  | See role as important |
|  |  | Need for more volunteers |
|  |  | More of a need for the service |
|  |  | Fear of fallout after pandemic |
|  |  | Challenge to maintain service |
|  |  | Commitment required in volunteering |

**Supplementary Material 2:**

*COREQ checklist for present study*

| **No. Item** | **Guide questions/description** | **Reported** |
| --- | --- | --- |
| **Domain 1: Research team and reflexivity** | | |
| *Personal characteristics* | | |
| 1. Interviewer/facilitator | Which author(s) conducted the interview or focus group? | Aoife Cooney |
| 1. Credentials | What were the researcher’s credentials? *E.g., PhD, MD* | BA Psychology  MSc Psychology & Wellbeing student |
| 1. Occupation | What was their occupation at the time of the study? | Student |
| 1. Gender | Was the researcher male or female? | Female |
| 1. Experience and training | What experience or training did the researcher have? | Prior experience of quantitative research as part of undergraduate degree, trained as a crisis line volunteer |
| *Relationship with participants* | | |
| 1. Relationship established | Was a relationship established prior to study commencement? | No, participants were not known to the researcher |
| 1. Participant knowledge of the interviewer | What did the participants know about the researcher? *E.g., personal goals, reasons for doing the research* | Participants were aware of the nature of the research study and that the project was being conducted as part of a Master’s degree |
| 1. Interviewer characteristics | What characteristics were reported about the interviewer/facilitator? *E.g., bias, assumptions, reasons and interests in the research topic* | Participants were informed that the interviewer is completing a Master’s degree |
|  | | |

**Supplementary Material 2:**

*COREQ (continued)*

| **No. Item** | **Guide questions/description** | **Reported** |
| --- | --- | --- |
| **Domain 2: Study design** | | |
| *Theoretical framework* | | |
| 1. Methodological orientation and Theory | What methodological orientation was stated to underpin the study? *E.g., grounded theory, discourse analysis, ethnography, phenomenology, content analysis* | Reflexive thematic analysis (Braun & Clarke, 2006; Braun & Clarke, 2019) |
| *Participant selection* | | |
| 1. Sampling | How were participants selected? *E.g., purposive, convenience, consecutive, snowball* | Convenience sampling |
| 1. Method of approach | How were participants approached? *E.g., face-to-face, telephone, mail, email* | Email |
| 1. Sample size | How many participants were in the study? | 13 |
| 1. Non-participation | How many people refused to participate or dropped out? Reasons? | One participant declined to participate after receiving the plain language statement and consent form, providing a reason of being unaware of the interview aspect of the study |
| *Setting* | | |
| 1. Setting of data collection | Where was the data collected? *E.g., home, clinic, workplace* | Home via Zoom (in line with COVID-19 guidelines at the time) |
| 1. Presence of non-participants | Who was present besides the participants and researchers? | Nobody else was present |
| 1. Description of sample | What are the important characteristics of the sample? *E.g., demographic data, date* | Individuals fully trained and actively volunteering with Samaritans branches in Ireland; full demographic data are provided on pages 22-23 |
| *Data collection* | | |
| 1. Interview guide | Were questions, prompts, guides provided by the authors? Was it pilot tested? | Yes, an interview schedule was developed prior. Pilot testing occurred with two additional Samaritans volunteers |

**Supplementary Material 2:**

*COREQ (continued)*

| **No. Item** | **Guide questions/description** | **Reported** |
| --- | --- | --- |
| 1. Repeat interviews | Were repeat interviews carried out? If yes, how many? | No |
| 1. Audio/visual recording | Did the research use audio or visual recording to collect the data? | Audio recording |
| 1. Field notes | Were field notes made during and/or after the interview or focus group? | No |
| 1. Duration | What was the duration of the interviews or focus group? | Interviews ranged in duration from |
| 1. Data saturation | Was data saturation discussed? |  |
| 1. Transcripts returned | Were transcripts returned to participants for comment and/or correction? | No, due to the time constraints on the study as a Master’s degree project. Participants were informed that they could request a copy of the transcript if they wished, however no participant made that request. |
| **Domain 3: Analysis and findings** |  |  |
| *Data analysis* |  |  |
| 1. Number of data coders | How many data coders coded the data? | One |
| 1. Description of the coding tree | Did authors provide a description of the coding tree? | Yes |
| 1. Derivation of themes | Were themes identified in advance or derived from the data? | Derived from the data; an inductive approach was taken |
| 1. Software | What software, if applicable, was used to manage the data? | NVivo (release 1.5.1 (940) for Windows) |
| 1. Participant checking | Did participants provide feedback on the findings? | No (refer back to item 23) |

**Supplementary Material 2:**

*COREQ (continued)*

| **No. Item** | **Guide questions/description** | **Reported** |
| --- | --- | --- |
| *Reporting* |  |  |
| 1. Quotations presented | Were participants quotations presented to illustrate the themes/findings? Was each quotation identified? *E.g., participant number* | Yes. Quotes were identified using participant pseudonyms to protect anonymity |
| 1. Data and findings consistent | Was there consistency between the data presented and the findings? | Yes |
| 1. Clarity of major themes | Were major themes clearly presented in the findings? | Yes |
| 1. Clarity of minor themes | Is there a description of diverse cases or discussion of minor themes? | Yes |

*Note:* Tong, A., Sainsbury, P., & Craig, J. (2007). Consolidated criteria for reporting qualitative research (COREQ): A 32-item checklist for interviews and focus groups. *International Journal for Quality in Health Care, 19*(6), 349-357. <https://doi.org/10.1093/intqhc/mzm042>
